# Interactions of the Invasive Fruit Flies *Drosophila suzukii*, *Zaprionus indianus*, *Zaprionus tuberculatus* and *Ceratitis capitata* with Their Hosts in the Brazilian Pampa Biome

**DOI:** 10.3390/insects16121285

**Published:** 2025-12-18

**Authors:** Karina Jobim, Bárbara Rafaela da Rosa, Pedro da Luz Kaster, Sergio Marcelo Ovruski, Flávio Roberto Mello Garcia

**Affiliations:** 1 Programa de Pós-Graduação em Fitossanidade, Departamento de Ecologia, Instituto de Biologia, Zoologia e Genética, Universidade Federal de Pelotas, Pelotas 96010-610, RS, Brazil; karina.pinto@ufpeel.edu.br; 2 Laboratório de Ecologia de Insetos (LABEI), Instituto de Biologia, Universidade Federal de Pelotas, Pelotas 96010-610, RS, Brazil; barbara.rosa@ufpel.edu.br (B.R.d.R.); pedro.kaster@ufpel.edu.br (P.d.L.K.); 3 Pilot Plant of Industrial Microbiological and Biotechnology Processes (PROIMI-CONICET), Biological Control Department, Avda. Belgrano and Pje. Caseros, San Miguel de Tucumán 4000, Argentina; sovruski@conicet.gov.ar

**Keywords:** community ecology, Drosophilidae, fruit fly abundance, host plant range Tephritidae, trophic interactions

## Abstract

Several fruit fly species in the Drosophilidae and Tephritidae families are classified as invasive pests worldwide. However, limited information is available on the occurrence of those dipteran species, their interactions, and their host plants in the Pampa Biome of southern Brazil. Therefore, we investigated interactions between invasive fruit flies and host plants. After two years (2022–2023) of fruit surveys, 3802 fruits from 16 plant species were harvested in the Pampa Biome’s urban and rural areas. The collected fruits were conditioned in the laboratory. Emerged adult flies were identified and associated with the respective fruit species. The most abundant species detected were *Zaprionus indianus* (Gupta), followed by *Zaprionus tuberculatus* (Malloch), *Drosophila suzukii* (Matsumura), and *Ceratitis capitata* (Wiedemann). The invasive drosophilids *D. suzukii*, *Z. indianus* and *Z. tuberculatus* interacted with 10 host plant species each, whereas *C. capitata* was associated with seven host species. *Zaprionus indianus* showed the highest infestation rate on fruits collected from the ground, whereas *D. suzukii* had the highest infestation rate in fruits attached to plants. New host plants were identified for *Z. tuberculatus* and *C. capitata*. The findings of this study provide a valuable framework for more effective management and control strategies.

## Simple Summary

Understanding the interactions among insects, their host plants, and natural enemies is an important challenge in community ecology. Such biological interrelationships help to understand how these insects compete for resources, with a significant impact on the structure and dynamics of the ecosystem. Thus, tephritid and drosophilid fruit flies are an interesting group of phytophagous insects to target for study. This study focused on assessing interactions and shared resources among invasive fruit fly species in the Pampa Biome of southern Brazil. Native and exotic fruit surveys were conducted during two consecutive years in highly disturbed environments in this region. Four exotic fruit fly species interacting with each other were identified in the region, *Drosophila suzukii*, *Zaprionus indianus*, *Zaprionus tuberculatus* and *Ceratitis capitata*. In addition, native Neotropical *Anastrepha* species were also identified. Notably, *D. suzukii* showed success and abundance when feeding on fruits collected directly from plants, followed by *C. capitata*. Both *Zaprionus* species were primarily collected from fallen fruits. The study provides valuable insights into hosts, abundance, and competition among the four invasive species, which are helpful for the development of future integrated pest management programs based on eco-friendly strategies.

## 1. Introduction

A relevant issue in ecological studies of exotic fruit fly pests is understanding the abundance of their populations and their interactions with native and exotic host plants within the invaded area. In this regard, the Brazilian Pampa Biome is of particular interest for the presence of invasive frugivorous insects and the potential interactions between them and the local ecosystem [1]. The Brazilian Pampa, also known as the Uruguayan Savanna ecoregion, is located in the northern part of the Río de la Plata region and spans 193,383 km^2^, representing 2.3% of Brazil’s territory [2]. The landscape features a mosaic of grasslands, various types of scrublands, low forests, and gallery forests [3]. Only 40% of the original vegetation remains in this ecosystem, mainly caused by deforestation. Invasive plant species have been introduced due to international trade between Brazil and other countries [4]. Approximately 40% of fruit species found in this biome are classified as small, and are mostly consumed fresh [5].

Fruit cultivation is an economically and socially relevant activity in this region, but it is threatened by persistent phytosanitary challenges caused by pest insects. The potential impact of this issue is significant, since many of the insect species classified as invasive pests are established in Brazil, such as fruit flies of the families Drosophilidae and Tephritidae [6,7]. Among the invasive flies considered pests are *Drosophila suzukii* (Matsumura), the spotted-wing Drosophila [8]; *Zaprionus indianus* (Gupta), the African fig fly [9]; *Zaprionus tuberculatus* (Malloch), the vinegar or pomace fly (all Drosophilidae); and *Ceratitis capitata* (Wiedemann), the Mediterranean fruit fly (Tephritidae), all of which are established in Brazil [10]

The invasive species *D. suzukii* and *C. capitata* are native to Southeast Asia and Sub-Saharan Africa, respectively. They are serious primary pests that have spread throughout the Americas, Asia, Africa, and Oceania [11,12], whereas *Z. indianus* and *Z. tuberculatus*, of Afrotropical origin, are now present in the Americas, Asia and Europe [10,13,14].

*Ceratitis capitata* has a wide host range, with over 350 host plants from 65 families in Latin America [11], whereas *D. suzukii* has only been recorded on 64 host species from 25 families [12]. Both fruit fly species can oviposit in healthy fruits still on the plant because they have robust ovipositors [11,12]. *Zaprionus indianus* is considered only to be a primary pest of figs, but it has been recorded from 80 hosts in over 30 families [15], while *Z. tuberculatus* has 49 host plants belonging to 11 botanical families [16]. Among these four invasive dipteran species, the first to establish itself in Brazil was *C. capitata* in 1901 [17], followed by *Z. indianus* in 1999 [9], *D. suzukii* in 2013 [8], and *Z. tuberculatus* in 2020 [18]. *Ceratitis capitata* currently has the most extensive distribution in Brazil, occurring in 24 states [19], followed by *Z. indianus*, which occurs in 17 states [20], *D. suzukii* in 8, and *Z. tuberculatus* in 7 [10].

An understanding of the interactions among invasive fruit fly species, their host plants, and parasitoids in recently invaded regions is essential to support integrated pest management programs with an area-wide approach [21]. Therefore, we aimed to identify the host plant ranges of these invasive fruit fly species, evaluate preferred hosts, analyze interactions among the four dipteran species, and determine whether there is resource competition or coexistence in the same niche.

## 2. Materials and Methods

### 2.1. Fruit Sampling

Fruit surveys were performed between January 2022 and December 2023, in several urban and rural areas of the city of Pelotas, Rio Grande do Sul State, southern Brazil. Fruits were taken to the Insect Ecology Laboratory (LABEI, Portuguese acronyms) at Universidade Federal de Pelotas. Harvesting was carried out on native and exotic plants, collecting approximately 30 fruits per plant, including fallen and/or attached fruits. These fruits were kept under laboratory conditions (24 ± 2 °C, 70 ± 10% RH, and a photoperiod of 12:12 h (L:D). All fruits were collected from orchards, urban areas, yards, or parks (Table 1).

### 2.2. Fruit Packaging and Insect Emergence

All fruits collected were weighed individually on an analytical scale and placed in transparent containers covered with voile fabric. Container size varied according to fruit size. A 2 cm layer of extra-fine vermiculite was placed in each container to prevent fruit rotting and subsequent absence of pupae. The number of flies emerged per fruit species and per collecting area was recorded.

### 2.3. Insect and Plant Identification

*Drosophila suzukii* and *Z. indianus* were identified using the external morphological features detailed in a taxonomical key [22]. *Zaprionus tuberculatus* was identified based on the diagnostic characteristics described in the paper [18], and *C. capitata* was identified according to the diagnostic morphological characteristics described in the book [23]. Host plant species were identified based on morphological characteristics [24]. Adult voucher specimens were stored at the entomological collection of the Insect Ecology Laboratory at Universidade Federal de Pelotas.

### 2.4. Data Analysis

The infested fruit percentage (PIF) was calculated using the formula number of infested fruits × 100/total number of sampled fruits. In addition, infestation indices were calculated according to two equations: (1) (M/n), where M = number of emerged flies, n = total fruits infested, and (2) (M/PF), where M = number of flies emerged and PF = fruit weight [25].

An analysis was carried out using the interaction network building software R (R Core Team), version 4.4.0, through the package “Bipartide”. The “species level” function of the software allowed the description of the level of specialization of *D. suzukii*, *Z. indianus*, *Z. tuberculatus*, and *C. capitata* in relation to the hosts. In addition, it was possible to verify the host preference degree of the fly species within a weighted network, taking into account information such as abundance and distribution of interactions among available hosts [26].

## 3. Results

A total of 3802 fruits from 16 species, in seven botanical families, were collected, totaling 35,882 kg (Table 1). Out of this total, 3109 fruits were collected from the ground: *Butia capitata* (Mart.) Becc. (Arecaceae), *Morus nigra* Linnaeus, (Moraceae), *Acca sellowiana* (Berg) Burret (Myrtaceae), *Campomanesia xanthocarpa* (Mart.) O. Berg (Myrtaceae), *Eugenia aggregata* (Vell.) Kiaersk (Myrtaceae), *Eugenia uniflora* Linnaeus (Myrtaceae), *Psidium cattleianum* Sabine, (Myrtaceae), *Psidium guajava* Linnaeus (Myrtaceae), *Syzygium cumini (*Linnaeus) Skeels (Myrtaceae), *Passiflora caerulea* Linnaeus (Passifloraceae), *Prunus persica (*Linnaeus) Batsch (Rosaceae), *Citrus reticulata* Blanco (Rutaceae), and *Citrus sinensis* Linnaeus (Rutaceae).

A total of 693 fruits were also collected from plants of eight species from five botanical families: *E. aggregata* (Myrtaceae), *E. uniflora* (Myrtaceae), *P. cattleianum* (Myrtaceae), *P. caerulea* (Passifloraceae), *Eriobotrya japonica* (Thunberg) Lindley (Rosaceae), *Rubus fruticosus* Linnaeus (Rosaceae), *C. sinensis* (Rutaceae), and *Vitis labrusca* Linnaeus cv Isabel (Vitaceae).

From a total of 16,855 drosophilids that emerged, 92% (15,560) were recorded from fallen fruits, of which 772 individuals were *D. suzukii* (4.8%), 8247 *Z. indianus* (53.1%), 3343 *Z. tuberculatus* (21.5%), and 3198 belonged to other drosophilid species (20.6%).

Regarding tephritid species sampled, a total of 1338 individuals were found from fallen fruits, of which 239 were *C. capitata* (26.2%) and 672 belonged to the genus *Anastrepha* (73.8%), particularly the *Anastrepha* complex. Among the species *B. capitata* and *C. sinensis*, there was no emergence of individuals in the *Anastrepha* genus.

According to the PIF (but only with regard to invasive fly species), the highest infestation percentage in fruits collected from the ground were *B. capitata* (89.5%) and *A. sellowiana* (87.8%), whereas the lowest infestations in fallen fruits were *P. caerulea* (8.0%), *C. xanthocarpa* (8.7%), and *M. nigra* (12.7%).

In fruits collected directly from plants, a total of 1325 drosophilid individuals were found, of which 712 were *D. suzukii* (4.8%), 261 *Z. indianus* (53.1%), 67 *Z. tuberculatus* (5.1%), and 285 belonging to other drosophilid species (21.5%). In addition, a total of 50 individuals were identified as *C. capitata* (11.7%), and 377 belonged to the *Anastrepha* genus (88.3%). In fruits still on the plants, the highest infestations occurred in *E. aggregata* (86.5%) and *R. fruticosus* (85.1%). *Drosophila suzukii* mainly attacked both host plants. The lowest infestation rates were found in *E. japonica* (3.0%), *C. sinensis* (23.3%), and *V. labrusca* (26.3%) (Table 2).

The PIF index (Table 2) for *D. suzukii* was high in fallen fruit species such as *E. aggregata* (63.8%) and *E. uniflora* (26.9%). In fruits still on plants, *D. suzukii* had the highest infestation rates in *E. aggregata* (86.5%), *E. uniflora* (80.0%), and *R. fruticosus* (61.5%). When comparing the number of flies per fruit (M/n), *D. suzukii* had the minimum of one insect per fruit in all ten hosts. According to the M/n index (flies/single fruit), the highest values found for *D. suzukii* were in hosts collected from the plant, with 6.2, 5.1, and 2.5 flies per fruit in *R. fruticosus*, *E. aggregata,* and *V. labrusca,* respectively. This was related to the number of flies per kg of fruit, as it involves fruit size and the available host mass for insect development. According to the M/PF index (flies/kg fruit), *D. suzukii* had the highest values in fallen fruit from *E. uniflora* (773 flies) and *E. aggregata* (249 flies). The M/PF index for *E. aggregata* infested by *D. suzukii* was 7 times higher in fruits still on the plant than in fallen fruits, recording 1739 flies per kg. Furthermore, *among D. suzukii* host plants, the highest index was observed for *V. labrusca* (940 flies) and *E. uniflora* (930 flies). In *E. uniflora*, the M/PF index for *D. suzukii* was only slightly higher in fruits still on the plant compared to fallen fruits.

The highest infestation percentage (PIF) (Table 2) recorded for *Z. indianus* was by *B. capitata* (82.7%). This invasive drosophilid species had more than 10 insects per fruit in three hosts collected from the ground, namely *B. capitata* (15.1), *P. guajava* (14.6), and *C. sinensis* (13.8). However, in fruits still on the plant, *Z. indianus* had only a high M/n index in *C. sinensis* (16.3). The highest M/PF indices recorded for *Z. indianus* were in *E. uniflora* (3192 flies/kg) and *B. capitata* (1323) fruits, collected from the ground, and in *E. uniflora* (1.866) and *P. cattleianum* (1.320) fruits surveyed from plants.

The highest percentages of infestation (Table 2) by *Z. tuberculatus* were found in *C. reticulata* (71.4%), *C. sinensis* (42.3%), *B. capitata* (15.1%), and *P. guajava* (14.6%) fruits collected from the ground. The highest M/n indices were found in fallen fruit of *C. sinensis* (18.4 flies) and *C. reticulata* (13.4), and also in *E. uniflora* (3.9) fruits, but collected from the plant. The highest M/PF indices recorded for *Z. tuberculatus* were in *E. uniflora*, both from the ground (2392 flies/kg) and from the plant (2595).

The highest infestation rates by *C. capitata* were observed in *C. sinensis* fruits from the ground (15.9%) and from the plant (16.7%). Infestation percentages did not exceed 10% in the other hosts where *C. capitata* was found (Table 2). The highest M/n index was also recorded for both *C. sinensis* fallen fruit (6.1) and fruit taken from the plant (9). The highest M/PF indices recorded for *C. capitata* were in *P. persica* (692 flies/kg), *P. cattleianum* and *E. uniflora* (303) fruits collected from the ground, and in *P. cattleianum* (413) fruits collected from the plant.

The highest percentages of infestation by *Anastrepha* spp. were in *P. guajava* (100%), *P. caerulea* (92.0%), and *A. sellowiana* (80.5%) fruits collected from the ground, and in *P. caerulea* fruits taken from the plant (66.1%). M/n indices higher than two individuals per fruit were only recorded in fallen *P. guajava* fruits (3.2 flies) and in *P. caerulea* fruits collected from the plant (3.1). The highest M/PF indices recorded for *Anastrepha* spp. were recorded in *C. xanthocarpa* (761 flies/kg) and *E. uniflora* (550) fruits collected from the ground, and in *E. aggregata* fruits collected from the plant (520).

We also found two new hosts for the Mediterranean fruit fly, *C. capitata*, where both *B. capitata* and *P. caerulea* have not yet been locally registered as hosts. In addition, two hosts (*P. cattleianum* and *E. uniflora*) are reported here for the first time for the species *C. capitata* in the Pampa Biome. Three new hosts were also identified for the species *Z. tuberculatus*, wild: *C. reticulata*; *R. fruticosus* and *S. cumini*.

**Figure 1:**
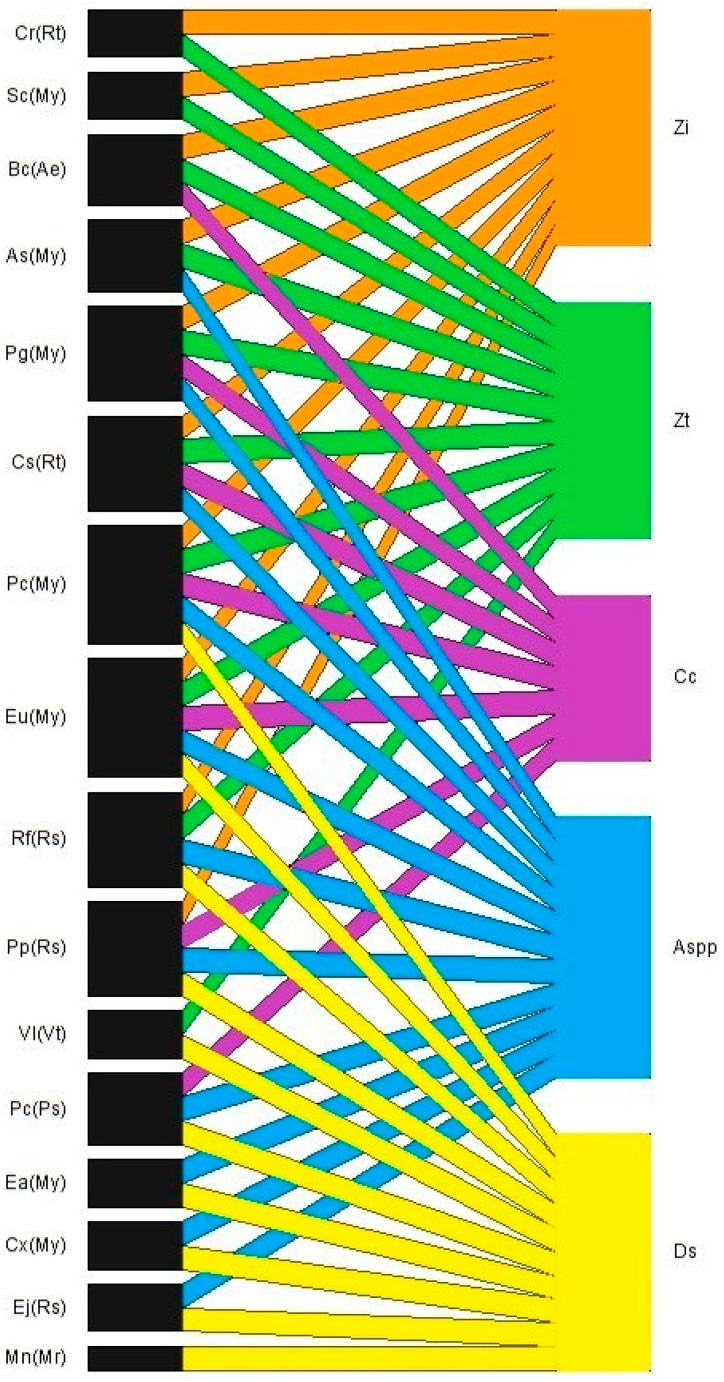
Network of interactions between host species and invasive fruit flies occurring in the Brazilian Pampa Biome. Zi = *Zaprionus indianus*; Zt = *Zaprionus tuberculatus*; Cc = *Ceratitis capitata*; Aspp = *Anastrepha* Spp.; Ds = *Drosophila suzukii*; Cr(Rt) = *Citrus reticulata (*Rutaceae); Sc(My) = *Syzygium cumini* (Myrtaceae); Bc(Ae) = *Butia capitata* (Arecaceae); As(My) = *Acca sellowiana* (Myrtaceae); Pg(My) = *Psidium guajava* (Myrtaceae); Cs(Rt) = *Citrus sinensis* L. osbeck (Rutaceae); Pc(My) = *Psidium cattleianum* (Myrtaceae); Eu(My) = *Eugenia uniflora* (Myrtaceae); Rf(Mr) = *Rubus fruticosus* (Rosaceae); Pp(Rs) = *Prunus persica* (Rosaceae); Vl(Vt) = *Vitis labrusca* L. cv Isabel (Vitaceae); Pc(Ps) = *Passiflora caerulea* L. (Passifloraceae); Ea(My) = *Eugenia aggregata* (Myrtaceae); Cx(My) = *Campomanesia xanthocarpa* (Myrtaceae); Ej(Rs) = *Eriobotrya japonica* (Rosaceae); Mn(Mr) = *Morus nigra* (Moraceae).

Figure 1 shows the interaction network among the four identified invasive fruit fly species and their hosts.

Furthermore, we found that the network of interactions between hosts and flies was nested (16 × 6), as evidenced by the NODF values of 60.46, showing moderate to high nestedness, in which the species interact with their hosts, mostly with defined interactions and structures, as evidenced by their modularity Q 0.23, with a high degree of connectivity of the species in sharing the same host, obtaining a similar response through the null model analyses, thus validating the results obtained. Furthermore, the network revealed interaction with at least one exotic fruit fly species in all sampled host plants.

## 4. Discussion

In this study, interactions between host plants and the invasive fruit fly species *D. suzukii*, *Z. indianus*, *Z. tuberculatu*s and *C. capitata* and the native *Anastrepha* spp. genus were recorded for the first time in the Brazilian Pampa Biome. In this context, our results are valuable ecological findings, as follows: (1) the high occurrence of the four invasive fruit fly species in native host fruits; (2) the high level of association among invasive fruit fly species with hosts belonging to the Myrtaceae family; (3) the difference in abundance and fruit infestation levels and host preference between the exotic primary pest species, *D. suzukii* and *C. capitata*; (4) the prevalence of *D. suzukii* in fresh fruit sampled from the plant relative to fruits collected from the ground; (5) the coexistence of *D. suzukii* and *C. capitata* in native hosts; (6) the finding of new medfly host plants for Brazil and for the Brazilian Pampa Biome; (7) the high abundance and incidence of the invasive, secondary pest species, *Z. indianus* and *Z. tuberculatus*, in most sampled fruits; and (8) the coexistence in a high number of fruits, mainly native, among native species of the *Anastrepha* genus and invasive, harmful fruit fly species, particularly *D. suzukii* and *C. capitata*.

The first finding revealed that each invasive fruit fly species infested 50% of the total native host fruits collected in the region, which shows the large niche available for invasive species, and the possibilities of interactions between such exotic fly species and hosts. Although part of the environment where fruits were collected is anthropized, with habitats having a high level of disturbance, they are not under any phytosanitary control measures, thus favoring the multiplication and survival of pest species. Thus, one hypothesis is that these species may be using such spaces as refuge areas between harvests [7]. Interestingly, the most vulnerable host plant was the native *E. uniflora*, which was infested by all four invasive dipteran species, indicating the availability of several resources for the population growth of invasive dipteran species in urban areas, providing refuge for the proliferation of exotic fruit fly species between harvests [27,28].

The second finding indicated that although *D. suzuk*ii, *Z. indianus*, *Z. tuberculatus* and *C. capitata* shared different host plants, only in two native Myrtaceae species (*E. uniflora* and *P. cattleianum*) were the four exotic flies found concurrently interacting. Among the remaining five Myrtaceae species infested by invasive flies, simultaneous coexistence between *C. capitata* and both *Zaprionus* species was only recorded in *P. guajava*. Myrtaceae fruits have a strong aromatic odor, particularly produced by phenolic compounds, which make them more attractive and susceptible to fruit fly infestation [29].

The third finding revealed higher abundance and infestation levels of *D. suzukii* than of *C. capitata* at the sampling sites where both fruit flies were found cohabiting. In addition, *D. suzukii* was the only primary pest species to infest *C. xanthocarpa*, *E. aggregata*, *E. japonica*, and *M. nigra*, and it was found only associated with native *Anastrepha* spp. on these hosts. In contrast, *C. capitata* exhibited a habitat-sharing behavior, because it was always found on fruit hosts interacting with the other three invasive fly species, and/or with *Anastrepha* spp. This suggests increased interspecific competition between *C. capitata* and the remaining invasive flies for space—such as oviposition and mating sites—and for food resources, as occurs when fruit fly species cohabit in a small area, sharing hosts [30]. Also, *C. capitata* was the fruit fly species with the fewest host interactions, involving only seven plant species. This was probably related to the host preference of *C. capitata*, since *Citrus* spp. are greatly preferred by this exotic fly species [31]. In the current study, this host preference was confirmed: *C. capitata* mainly infested *C. sinensis* fruits, the host with the highest infestation level. *Drosophila suzukii* showed a strong preference for small, reddish, and purple fruits, on which it had the highest infestation levels and prevailed over the other invading fruit flies. Such information supports previous studies of *D. suzukii* host preference, although there are also reports of infestation in other hosts, such as persimmons, figs, apples, pears, nectarines and peaches [32,33,34]. In this study, *D. suzukii* was also found in *P. persica* but with a lower infestation level than in host species with smaller fruits than peaches.

The fourth finding verified that *D. suzukii* was most abundant in fruit collected from the plant. This behavior of *D. suzukii* females is probably associated with avoiding egg-laying on/in fruits already inhabited by other drosophilids, mainly saprophytic species, thereby preventing resource competition [35]. However, the impact of interspecific interactions may vary depending on the species coexisting with *D. suzukii* [36,37]. In addition, *D. suzukii* is able to drop heterospecific chemical signals on substrates (fruits) during oviposition, behavior that is highly important to facilitate aggregation and mating [38,39,40]. Such aggregation pheromones provide a signal to other *D. suzukii* females about the substrate quality, which allows them to have an advantage over other competing frugivorous fly species [41]. Thus, *D. suzukii* females do not avoid substrates that already contain eggs of the species itself [42]. This was verified in this study: *D. suzukii* had a higher incidence on all fruit species sampled from the plant than on hosts collected from the ground, which are more susceptible to infestation by opportunistic, saprophytic fly species that tend to prefer overripe or rotten fruits.

The fifth finding highlighted the essential role of native fruits as propagation resources of the two primary pest species in the region. Three of the four host species on which *D. suzukii* and *C. capitata* were found cohabiting are native. Interestingly, both fruit fly species coexisted in four host species, namely *P. caerulea*, *E. uniflora*, *P. cattleianum* (all natives), and *P. persica* (exotic), but with a predominance of the first pest fly species. In this context, about 40% of the hosts infested by *D. suzukii* were shared with *C. capitata*, whereas medflies coexisted with *D. suzukii* in 60% of the total number of hosts attacked by this native African fly species. Interestingly, the coexistence of *D. suzukii* and *C. capitata* on an introduced host species, *P. persica*, was previously reported in a highly disturbed secondary rainforest environment in northern Argentina [43]. Therefore, the results of the current study corroborate evidence from surveys in Argentina, suggesting that highly disturbed natural environments with a high diversity of host plants favor the coexistence and proliferation of *D. suzukii* and *C. capitata*. Both exotic fruit fly species exhibit high environmental plasticity, allowing these dipterans to exploit and thrive in diverse environments across several regions of the world [43]. This finding has important practical implications for the integrated management of both primary fruit fly pests. This is particularly relevant when biological control becomes a viable tool in areas of disturbed wild vegetation.

The sixth finding revealed two new host fruit species associated with *C. capitata* in Brazil, *B. capitata* and *P. caerulea*. Thus, with 116 host plants previously recorded in Brazil [44], this study increased the total number of medfly hosts to 118. In addition, the current study showed, for the first time, that both *P. cattleianum* and *E. uniflora* were infested by *C. capitata* in the Brazilian Pampa Biome. Therefore, the diversity of medfly hosts in this biome increased to 17 plant species, based on 13 host species previously reported for the region [44].

The seventh finding showed, in terms of overall abundance, that both *Z. indianus* and *Z. tuberculatus* were the most frequent of the four invasive fruit fly species identified in fruit samples, but with *Z. indianus* predominantly found in fallen fruit. Interestingly, both *Z. indianus* and *Z. tuberculatus* shared 80% of the total number of hosts on which the two drosophilid species were found. Both flies were simultaneously recovered from fallen fruits. Such data are in agreement with previous studies, which highlighted both *Z. indianus* and *Z. tuberculatus* as secondary pests that mainly infest damaged or decaying fruits, mostly on the ground [45,46]. However, *Z. tuberculatus* is also capable of laying eggs in intact fruits such as pears, strawberries, figs and pomegranates, making the species a potential threat due to the damage in can cause to fruit production in the areas where it establishes itself [47]. However, both invasive *Zaprionus* species may increase the damage caused in commercially valuable crop fruits by primary pest species, such as *D. suzukii* and *C. capitata*, since *Z. indianus* and *Z. tuberculatus* are opportunistic species [45,46,48]. In addition, *Z. indianus* was able to oviposit and develop in healthy strawberries grown in Brazil, but infestations were higher when the fruits were previously damaged [48]. Consequently, the present study revealed that 50% and 66% of the fruits infested with *D. suzukii* and *C. capitata*, respectively, and collected from the plant, were also infested with the two *Zaprionus* species, but mainly with *Z. indianus*. This suggests a possible interdependence between the invasive secondary pests and the primary pest species. Several studies have pointed out that *Drosophila suzukii* frequently shares the same niche with both *Zaprionus* species [13,37]. This study also revealed that *Z. tuberculatus* infested commercial fruit species, such as *V. labrusca*, *C. reticulata*, and *C. sinensis*, which are economically important for the Brazilian export and domestic markets [49]. Likewise, *C. sinensis* was highly infested by *C. capitata*, which probably favored *Z. tuberculatus* infestation. The high abundance of both *Zaprionus* species and their wide range of host species recorded in this study support the rapid adaptation to the environmental conditions and structural characteristics of highly disturbed habitats in the Pampa Biome, as reported in previous studies for both this biome and other Brazilian regions [50,51]. Some studies have already highlighted the importance of monitoring invasive species, including in anthropized urban environments [27,28] since some species, such as *Z. indianus* and *Z. tuberculatus*, although considered secondary pests, can increase the dam-age caused by primary species such as *D. suzukii* e *C. capitata*. The study also supports a higher abundance of *Z. indianus* on fruits of *B. capitata*, as previously reported in Brazil [52]. This host plant is, after the fig, the most preferred by *Z. indianus*. This invasive dipteran was also reported on small fruits of different plant species in southern Brazil [25], but with low infestation levels and preferences different from those observed in the present study. These differences are probably related to the structure and degree of disturbance of the studied habitats. Poorly disturbed natural habitats usually exhibit a higher diversity of available native hosts, but with a predominance of the *Anastrepha* genus [53]. With respect to *Z. tuberculatus*, this study revealed higher abundance in fruits of *C. reticulata*, *C. sinensis*, *B. capitata*, and *P. guajava*. However, *A. sellowiana*, *E. uniflora*, *M. nigra*, *P. cattleianum*, and *V. labrusca* L. cv Isabel have been reported as important hosts in different regions of Brazil [54,55,56,57,58].

The eighth finding revealed a high prevalence of *Anastrepha* spp. on native fruits, particularly those of the Myrtaceae family, which are still found in disturbed environments of the studied biome. Specimens of the *Anastrepha* genus were recovered in 86% of the total Myrtaceae species sampled in the current study. This is consistent with previous studies reporting a preference for *Anastrepha fraterculus* complex species on Myrtaceae fruit in America [58,59]. Interestingly, coexistence of native *Anastrepha* species with the invasive pests *D. suzukii* and *C. capitata*, both primary pests, was observed in 90% of plant species infested by any of the above exotic pests. This shows that the invasive flies in the region may interact with *Anastrepha* spp. in highly modified environments, consistent with findings in northern Argentina [43].

## 5. Conclusions

Our results showed that the combination of a highly disturbed environment and a high diversity of exotic and native fruit species has created a habitat where multiple fruit fly species, both invasive and native, can coexist. Apparently, coexistence between exotic primary pests and native *Anastrepha* pests does not constrain the proliferation of *D. suzukii* and *C. capitata*. However, they compete for fresh fruit as a source of egg-laying. In turn, opportunistic invasive species, such as *Z. indianus* and *Z. tuberculatus*, are prevalent in the anthropized systems of the Brazilian Pampa Biome, thriving on damaged fruit on the plant, but mainly on rotting or decaying fallen fruit. In this region, native host species play an important role in sustaining and increasing populations of such exotic flies during the fruit crop off-season. Therefore, both *D. suzukii* and *C. capitata*, serious pests of regional fruit production, have secure, well-resourced habitats in the region. Consequently, there is a need to develop sustainable, environmentally friendly alternatives for the integrated management of these fruit fly pests, reducing or replacing the use of chemical products. A viable and promising strategy is biological control through conservation and/or augmentation, since natural enemies with potential as biocontrol agents, such as parasitoids and predators, can be found in the ecosystems of the Pampa Biome [60]. In this regard, the findings of this study provide valuable, up-to-date information for developing a biological control program, offering insights into interactions between invasive fruit fly pests and host infestation patterns.

## Figures and Tables

**Table 1:** Host plants and location (geographic coordinates) of fruit collection areas. E = Exotic, N = native.

Host Plants	Abbreviation	Origin	Common Name	Coordinates *
**Arecaceae**				
*Butia capitata*	Bc(Ae)	N	Jelly palm	Area 1
**Moraceae**				
*Morus nigra*	Mn(Mr)	E	Black mulberry	Areas 2, 3, 4, and 5
**Myrtaceae**				
*Psidium cattleianum*	Pc(My)	N	Strawberry guava	Areas 6, 7, 8, and 9
*Eugenia aggregata*	Ea(My)	N	Cherry	Area 10
*Psidium guajava*	Pg(My)	N	Guava	Area 11
*Acca sellowiana*	As(My)	N	Feijoa	Area 10
*Campomanesia xanthocarpa*	Cx(My)	N	Guabiroba	Areas 9 and 10
*Syzygium cumini*	Sc(My)	E	Jamelão	Areas 12 and 13
*Eugenia uniflora*	Eu(My)	N	Surinam cherry	Areas 1, 10, 7, 14, 15, and 16
**Passifloraceae**				
*Passiflora caerulea*	Pc(Ps)	N	Blue passion flower	Areas 5 and 18
**Rosaceae**				
*Eriobotrya japonica*	Ej(Rs)	E	Loquat	Areas 8, 9 and 19
*Prunus pérsica var. nucipersica,*	Pp(Rs)	E	Nectarine	Area 16
*Rubus fruticosus*	Rf(Rs)	E	Blackberry	Area 19
**Rutaceae**				
*Citrus reticulata*	Cr(Rt)	E	Tangerine	Area 8
*Citrus sinensis*	Cs(Rt)	E	Sweet orange	Area 5
**Vitaceae**				
*Vitis labrusca* cv Isabel	Vl(Vt)	E	Fox grape	Area 9

* Coordinates (areas): (1) 31°45′40″ S, 52°19′51″ W; (2) 31°45′34″ S, 52°21′54″ W; (3) 31°45′38″ S, 52°21′40″ W; (4) 31°45′29″ S, 52°22′10″ W; (5) 31°45′24.6″ S, 52°18′47.9″ W; (6) 31°45′42″ S, 52°19′52″ W; (7) 31°45′19″ S, 52°22′41″ W; (8) 31°45′05.4″ S, 52°23′13.0″ W; (9) 31°36′37.7″ S, 52°29′53.7″ W; (10) 31°35′54″ S, 52°28′28″ W; (11) 31°44′47.1″ S, 52°22′31.7″ W; (12) 31°45′25.1″ S, 52°22′43.5″ W; (13) 31°45′41.9″ S, 52°19′55.3″ W; (14) 31°44′59″ S, 52°22′35″ W; (15) 31°45′35.0″ S, 52°21′54.5″ W; (16) 31°46′46″ S, 52°19′53″ W; (17) 31°44′58″ S, 52°22′40.7″ W; (18) 31°44′57″ S; 52°22′36″ W; (19) 31°44′56.2″ S, 52°22′25.6″ W.

**Table 2:** Percentage of infested fruits (PIF), number of flies per infested fruit (M/n), and number of flies per kg of fruit (M/PF) recorded for frugivorous drosophilid flies *D. suzukii* (Ds), *Z. indianus* (Zi), *Z. tuberculatus* (Zt), and tephritid flies *Anastrepha* spp. (Aspp) and *C. capitata* (Cc) in relation to their hosts found in the Pampa Biome, southern Brazil.

		PFI (%).					M/n					M/PF				
Origin	Host	Ds	Zi	Zt	Aspp	CC	Ds	Zi	Zt	Aspp	CC	Ds	Zi	Zt	Aspp	CC
Ground	Bc(Ae)	0.0	82.7	52.9	0.0	0.9	0.0	15.1	5.4	0.0	1.0	0.0	1323.5	469.8	0.0	103.7
	Mn(Mr)	9.3	0.0	0.0	0.0	0.0	1.2	0.0	0.0	0.0	0.0	777.1	0.0	0.0	0.0	0.0
	As(My)	0.0	17.1	7.3	80.5	0.0	0.0	3.3	4.3	1.7	0.0	0.0	153.8	184.4	72.4	0.0
	Cx(My)	5.4	0.0	0.0	8.1	0.0	1.3	0.0	0.0	1.5	0.0	574.7	0.0	0.0	761.5	0.0
	Ea(My)	63.8	0.0	0.0	28.0	0.0	1.5	0.0	0.0	1.4	0.0	249.6	0.0	0.0	105.6	0.0
	Eu(My)	26.9	17.8	9.2	15.7	0.2	1.7	7.1	4.9	1.1	1.0	773.2	3192.1	2392.7	550.7	303.7
	Pc(My)	1.6	34.1	6.3	35.0	6.3	1.4	4.7	2.1	1.2	1.8	251.8	1139.7	434.4	352.4	421.7
	Pg(My)	0.0	59.2	42.9	100.0	4.1	0.0	14.6	2.7	3.2	1.0	0.0	353.4	66.0	79.8	27.8
	Sc(My)	0.0	61.3	9.3	0.0	0.0	0.0	4.8	1.3	0.0	0.0	0.0	1001.8	265.0	0.0	0.0
	Pc(Ps)	8.0	0.0	0.0	92.0	0.0	1.5	0.0	0.0	1.3	0.0	244.4	0.0	0.0	216.9	0.0
	Pp(Rs)	1.4	11.3	0.0	4.2	8.5	1.0	2.5	0.0	1.0	1.5	58.4	849.1	0.0	70.9	692.6
	Cr(Rt)	0.0	57.1	71.4	0.0	0.0	0.0	8.3	13.4	0.0	0.0	0.0	176.5	244.6	0.0	0.0
	Cs(Rt)	0.0	54.8	48.7	0.5	15.7	0.0	13.8	18.4	1.0	6.1	0.0	158.7	217.9	10.6	62.1
																
Plant	Ea(My)	86.5	0.0	0.0	59.5	0.0	6.2	0.0	0.0	1.2	0.0	1739.1	0.0	0.0	520.7	0.0
	Eu(My)	80.0	28.0	36.0	28.0	0.0	1.8	2.9	3.9	1.0	0.0	930.7	1866.5	2595.2	433.8	0.0
	Pc(My)	0.0	31.1	0.0	19.7	3.3	0.0	7.5	0.0	1.3	2.0	0.0	1320.4	0.0	198.1	413.0
	Pc(Ps)	0.0	0.0	0.0	66.1	0.8	0.0	0.0	0.0	3.1	1.0	0.0	0.0	0.0	343.5	159.6
	Ej(Rs)	2.0	0.0	0.0	16.0	0.0	1.0	0.0	0.0	1.3	0.0	80.9	0.0	0.0	74.1	0.0
	Rf(Rs)	61.5	18.2	7.4	37.2	0.0	5.1	1.8	1.6	1.2	0.0	619.3	498.7	452.0	404.7	0.0
	Cs(Rt)	0.0	10.0	0.0	0.0	16.7	0.0	16.3	0.0	0.0	9.0	0.0	173.3	0.0	0.0	100.7
	Vl(Vt)	3.4	0.0	6.9	0.0	0.0	2.5	0.0	1.2	0.0	0.0	940.5	0.0	429.3	0.0	0.0

List of abbreviations: Ds = *Drosophila suzukii*; Zi = *Zaprionus indianu*s; Zt = *Zaprionus tuberculatus*; Aspp = *Anastrepha* Spp.; Cc= *Ceratitis capitata*; Bc(Ae) = *Butia capitata* (Arecaceae); Mn(Mr) = *Morus nigra* (Moraceae); As(My) = *Acca sellowiana* (Myrtaceae); Cx(My) = *Campomanesia xanthocarpa* (Myrtaceae); Ea(My) = *Eugenia aggregata* (Myrtaceae); Eu(My) = *Eugenia uniflora* (Myrtaceae); Pc(My) = *Psidium cattleianum* (Myrtaceae); Pg(My) = *Psidium guajava* (Myrtaceae); Sc(My) = *Syzygium cumini* (Myrtaceae); Pc(Ps) = *Passiflora caerulea* L. (Passifloraceae); Pp(Rs) = *Prunus persica* (Rosaceae); Cr(Rt) = *Citrus reticulata* (Rutaceae); Cs(Rt) = *Citrus sinensis* L. osbeck (Rutaceae); Ej(Rs) = *Eriobotrya japonica* (Rosaceae); Rf(Rs) = *Rubus fruticosus* (Rosaceae); Vl(Vt) = *Vitis labrusca* L. cv Isabel (Vitaceae).

## Data Availability

The datasets analyzed in the present study are available from the corresponding authors on reasonable request.
